# Role of salicylic acid in regulating ethylene and physiological characteristics for alleviating salinity stress on germination, growth and yield of sweet pepper

**DOI:** 10.7717/peerj.8475

**Published:** 2020-03-27

**Authors:** Wazir Ahmed, Muhammad Imran, Muhammad Yaseen, Tanveer ul Haq, Muhammad Usman Jamshaid, Shah Rukh, Rao Muhammad Ikram, Muqarrab Ali, Anser Ali, Mudassar Maqbool, Muhammad Arif, Mahmood Alam Khan

**Affiliations:** 1 Department of Soil and Environmental Sciences, MNS-University of Agriculture, Multan, Multan, Pakistan; 2 Institute of Soil and Environmental Sciences, University of Agriculture Faisalabad, Faisalabad, Pakistan; 3 Department of Agronomy, MNS-University of Agriculture, Multan, Multan, Pakistan; 4 Department of Agronomy, Ghazi University, Dera Ghazi Khan, Dera Ghazi Khan, Pakistan; 5 Institute of Plant Breeding and Biotechnology, MNS-University of Agriculture, Multan, Pakistan; 6 Department of Environmental Geosciences National Centre of Excellence in Geology University of Peshawar, Peshawar, Pakistan

**Keywords:** Antioxidant activity, Ethylene, Salicylic acid, Sweet pepper, Salinity, Fertilizer use efficiency

## Abstract

**Background:**

During a preliminary study, effects of 0, 20, 40, and 60 mM NaCl salinity were assessed on germination rate in relation to electrolyte leakage (EL) in sweet pepper. Results explored significant rises in ethylene evolution from seeds having more EL. It was, therefore, hypothesized that excessive ethylene biosynthesis in plants due to salinity stress might be a root cause of low crop productivity. As salicylic acid is one of the potent ethylene inhibitors, thus SA was used to combat effects of ethylene produced under salinity stress of 60 mM NaCl on different physiological and morphological characteristics of sweet pepper.

**Methodology:**

The effect of 0.05, 0.1, 0.2, 0.3, 0.4, 0.5 and 0.6 mM SA was evaluated on seed germination, growth and yield of sweet pepper cv. Yolo wonder at salinity stress on 60 mM NaCl. Seeds were primed with SA concentrations and incubated till 312 h in an incubator to study germination. Same SA concentrations were sprayed on foliage of plants grown in saline soil (60 mM NaCl).

**Results:**

Seeds primed by 0.2 to 0.3 mM SA improved germination rate by 33% due to suppression of ethylene from 3.19 (control) to 2.23–2.70 mg plate^−1^. Electrolyte leakage reduced to 20.8–21.3% in seeds treated by 0.2–0.3 mM SA compared to 39.9% in untreated seeds. Results also explored that seed priming by 0.3 mM improved TSS, SOD and chlorophyll contents from 13.7 to 15.0 mg g^−1^ FW, 4.64 to 5.38 activity h^−1^ 100 mg^−1^ and 89 to 102 ug g^−1^ compared to untreated seeds, respectively. Results also explore that SA up to 0.2 mM SA applied on plant foliage improved LAI (5–13%), photosynthesis (4–27%), WUE (11–57%), dry weight (5–20%), SOD activity (4–20%) and finally fruit yield (4–20%) compared to untreated plants by ameliorating effect of 60 mM NaCl. Foliar application of SA also caused significant increase in nutrient use efficiency due to significant variations in POD and SOD activities.

**Conclusion:**

Salicylic acid suppressed ethylene evolution from germinating seeds up to 30% under stress of 60 mM NaCl due to elevated levels of TSS and SOD activity. Foliar application of SA upgraded SOD by lowering POD activity to improve NUE particularly K use efficiency at salinity stress of 60 mM NaCl. Application of 0.2 and 0.3 mM SA emerged as the most effective concentrations of SA for mitigating 60 mM NaCl stress on different physiological and morphological characteristics of sweet pepper.

## Introduction

Sweet pepper (*Capsicum annuum L.*) is the most important crop in solanaceous family after potato and tomato and is a popular crop among farmers of peri-urban areas in Pakistan (Mahmood et al., 2017). It is generally grown worldwide for its high remunerative value. In such areas, bell pepper is frequently exposed to saline conditions brought by saline water, loaded with NaCl (Ityel et al., 2014; Piñero et al., 2014). *Capsicum annum* cv. Yolo wonder is a very disease resistant variety of bell pepper and popular among farmers for its high yielding potential (Caruso et al., 2019). However, under arid to semi-arid regions particularly in Pakistan, it is frequently exposed to salinity induced by irrigation which causes a significant decline in its yield (Mahmood et al., 2017).

Soil salinity is one of the most devastating environmental stresses, limiting crop productivity and quality especially in arid to semi-arid regions (Shrivastava & Kumar, 2015; Zörb, Geilfus & Dietz, 2019). Salinity impairs plant growth by inducing ion toxicity, physiological drought, oxidative stress and nutrient deficiency particularly K deficiency (Zörb, Geilfus & Dietz, 2019), and thus reduces yields of crops up to 80% (Panta et al., 2014). Scarcity of water, improper irrigation drainage and abuse use of chemical fertilizers have threatened agriculture by salinity. In several decades, efforts have been made to elucidate salinity stress on plants through breeding strategies and approaches. Nowadays, integrative approach got more significance to explore the intrinsic mechanisms for plant rapid responses and self-modulation of growth to cope with salinity stress.

Ethylene is a gaseous hormone which plays multiples roles in plant physiology depending on its levels in plant tissues. It was well documented that salinity triggers ethylene in plants enormously which enhanced ROS and inhibited growth (Steffens, 2014). Conclusively, tight control of ethylene homeostasis is critical for survival of plants under high salinity stress. We planned to use salicylic acid (SA) for ethylene hemostasis in sweet pepper plants under salinity stress of 60 mM NaCl.

Salicylic acid (SA) is also a gaseous signaling phytohormones, which ameliorates effects of salinity on growth and development of plants (El-Esawi et al., 2017). SA also regulates ion uptake and antioxidant defense for inducing salinity tolerance in plants (Jayakannan et al., 2015). Works reported on SA elucidated that ethylene levels declined in plants after SA application either by inhibiting ethylene forming enzyme (Yang & Hoffman, 1984) and/or by enhanced synthesis of polyamines like spermidine and spermine (Freschi, 2013). Ethylene homeostasis in plants by SA depends on SA concentrations and type of crop e.g., SA ameliorated salinity stress on wheat at 0.05 mM (Shakirova et al., 2003) while on maize at 0.1 mM (Khodary, 2004) and 0.5 mM (Nemeth et al., 2002). Similarly, 0.1 mM SA inhibited chlorophyll contents but 0.0001 to 0.01 mM SA showed an increase in chlorophyll contents by 2 to 3.5 folds, respectively (Cag et al., 2009). Besides ethylene inhibition, exogenous SA improved nutrient uptake, photosynthetic activities, carbohydrate metabolism in plants (Hamid, Ashraf & Arshad, 2008), and fruit quality parameters (Jayakannan et al., 2015).

Keeping in view the above discussion, this study was planned to evaluate the ameliorating effect of SA on germination, nutrient uptakes, photosynthesis, growth and fruit yield of sweet pepper cv. Yolo wonder under salinity of 60 mM NaCl.

## Material and Methods

### Experiments description

During a preliminary study, effects of 0, 20, 40, and 60 mM NaCl salinity were assessed on growth and yield of sweet pepper. Owing to 60 mM NaCl of salinity, we found brutal reductions in growth and yield of sweet pepper (Preliminary study part of Ph.D Thesis). The objective of the current studies was to alleviate detrimental effects of 60 mM NaCl salinity on morphological and physiological aspects of sweet pepper by SA and determine optimal dose of SA for homeostasis of ethylene levels in bell pepper Yolo Wonder. Consequently, treatments of 0.05, 0.1, 0.2, 0.3, 0.4, 0.5, 0.6 mM SA were used for seed priming and foliar application. Each treatment was repeated thrice during lab study and four times during pot study. The cultivar of sweet pepper used was Yolo Wonder (F-1 progeny with 85% germination percentage distributed by California Production Seeds treated by producer with Thiram 50 MP dyed fungicide).

After surface sterilization with ethanol (70%), seeds were soaked in different SA solutions and deionized water then dried. The 30 primed seeds were placed over three round Whatman filter paper No. 42 (Schleicher and Schuell) in transparent petri-plates, covered by lid fitted with rubber septa and then sealed tightly with silicone gel and Para film tape (Para film “M” Laboratory Film, Pechiney Plastic Packing, Chicago, IL 60631). In each plate, 7 ml of 60 mM NaCl solution was injected through rubber septa on the lid for inducing salinity stress. All the activities were performed in laminar flow hood. After injecting water and sealing, all the plates were shifted to an incubator (Sanyo MIR 253) at 25 ± 2 °C day/night temperature with 14 h photoperiod using cool white fluorescent light.

Germinated seeds were counted at an interval of 24 h of incubation. At 312 h of incubation, the gas samples taken from each plate were analyzed for ethylene. At 312 h of incubation, seedling leaves were also sampled randomly for chlorophyll a + b and SA assay and stored at −80 °C. Seedling growth was estimated on the basis of root and shoot length and weight.

### Pot trial

A pot study was also conducted with the objective to test the efficiency of SA spayed on foliage for alleviating salt stress of 60 mM NaCl on growth and yield of sweet pepper. For this purpose, thirty days old plants were transplanted in pots. The soil was sandy clay loam, alkaline (pH 8.0), deficient in total N (0.03%) and available P (5.06 mg kg^−1^ soil). An optimal supply of N (30 mg kg^−1^ soil), P (60 mg kg^−1^ soil) and K (80 mg kg^−1^ soil) was maintained using urea, diammonium phosphate, and sulfate of potash, respectively. After 15 days of transplanting, treatments of SA were sprayed on plants whereas the next spray of SA treatments was carried out 30 days after transplanting. To nullify the effect of spray, deionized water was also sprayed on plants in control. Each pot was irrigated at or near field capacity by maintaining EC at 6 dS m^−1^. All plant protection measures were adopted throughout the entire crop period.

Growth-related morphological parameters such as number of branches, number of flowers, fresh and dry weight (g), and leaf area (cm^2^) were noted at 50 days after transplanting. Leaves were also sampled randomly for mineral and biochemical analysis. Fully grown fruits were picked and weighed at maturity. Leaf area was measured using a leaf area meter and then leaf area index (LAI) was also calculated. For assessing variations in chlorophyll contents, portable Chlorophyll meter (SPAD-502) was used.

### Estimation of physiological processes

Infrared Gas Analyser (IRGA, model ADC, Bioscientific Ltd., England) was used to measure photosynthetic rate (P_*N*_), Stomatal conductance (g_s_) and intercellular CO_2_ concentration (C_i_) in fully expanded new leaves at 1100–1200 h when above the plant canopy photosynthetic active radiations (PAR; 1,060 µmol m^−2^ s^−1^) were present. The inside temperature of the leaf cuvette was set at 30 ± 2 °C. The light responses curves were carried out at ambient CO_2_ concentrations (300–350 µmol mol^−1^). Instantaneous water use efficiency (WUE) was computed as the ratio of photosynthetic rate to transpiration (Polley, 2002).

### Determination of biochemical attributes

The ethylene was analyzed from gas samples collected from Petri plates using Gas Chromatography (Shimadzu, 2010) fitted with flame ionization detector (FID) and a capillary column i.e., Porapak Q 80-100 (Khalid et al., 2006).

For assay of SA in seedling, Trichloroacetic acid (TCA) based method earlier reported by Zhang et al. (2003) was used. Salicylic acid from seedling was extracted using ethyl ether extract. The residue obtained from ethyl ether exact was dissolved in a 0.02 M ammonium acetate in 50% methanol (pH 3.2). Then, 10 mL of extract + 10 mL of NH_4_Fe (SO_4_)_2_ was mixed and shaken well and centrifuged at 2,500 rpm for 3 min. The reading of color was recorded at a wavelength of 540 nm on double beam spectrophotometer. De-ionized water instead of the sample was run as blank.

Superoxide dismutase (SOD) activity was measured using the photochemical method (Winterbourn et al., 1975). Assays were carried out under illumination. One unit SOD activity is defined as the amount of enzyme required to cause 50% inhibition of the rate of p-nitro blue tetrazolium chloride reaction at 560 nm.

Lipid peroxidation (POD) was assayed by spectrophotometry using TBA-MDA assay (Minotti & Aust, 1987). Lipid peroxides were extracted with 5 mL of 5% (w/v) metaphosphoric acid and 100 mL of 2% (w/v in ethanol) butyl hydroxytoluene. An aliquot of the supernatant was reacted with thiobarbituric acid at 95 °C and cooled to room temperature. The resulting was extracted with 1-butanol.

Total soluble sugar contents (TSS) were also assayed using the method of Dong et al. (2001). One to two drops of the supernatant, as prepared above, was placed on the prism of the digital refractometer (Model ATAGO, Japan) and TSS was reported as °Brix (noted in percentage).

Chlorophyll pigments (a, b) from leaves were extracted by the help of acetone (85%), measured at 644 and 663 nm by spectrophotometer (Metzner, Rau & Senger, 1965). Finally, chlorophyll was calculated using the following equations:

\begin{eqnarray*}& & \text{Chlorophyll-a}=10.3 A663-0.918 A644 (\mathrm{\mu }{\text{g g}}^{-1}) \end{eqnarray*}

\begin{eqnarray*}& & \text{Chlorophyll-b}=19.7 A644-3.870 A663 (\mathrm{\mu }{\text{g g}}^{-1}). \end{eqnarray*}

For electrolyte leakage (EL), uniform leaf discs were added to 5 mL of deionized water in a test tube which was shaken for 4 h at 25 ± 1 °C. The EC of the solution containing leaf discs was measured before and after autoclave and finally, electrolyte leakage was calculated by using the formula:

\begin{eqnarray*}& & EL(\text{%})= \frac{\text{EC of solution before autocalve}}{\text{EC of solution after autocalve}} \times 100 \end{eqnarray*}

### Nutrient analysis, uptake and use efficiency

Plant samples were grounded and digested using 20 mL of H_2_SO_4_ (conc.) and 8 g digestion mixture (K_2_SO_4_: FeSO_4_:CuSO_4_ = 10:1:0.5) for each sample (Wolf, 1996). Nitrogen was analyzed according to Jackson (1962) while phosphorus was determined using vanadate-molybdate spectrophotometric procedure (Jones, Wolf & Mills, 1991). For potassium, the method given by Chapman & Pratt (1961) was followed. Nutrient uptake and use efficiency were calculated using the following formula:

\begin{eqnarray*}& & \text{Nutrient uptake}= \frac{\text{Nutrient conc.} \text{in plant}(\text{%})\times \text{oven dried weight}}{100} \end{eqnarray*}

\begin{eqnarray*}& & \text{Agronomic NUE}(\mathrm{g}/\mathrm{g})= \frac{\text{Yield of treated plants}-\text{Yield of control plant}}{\text{Nutrient applied}} \end{eqnarray*}

\begin{eqnarray*}& & \text{Recovery efficiency}= \frac{\text{Nutrient uptake increment per unit nutrient applied}}{\text{Nutrient applied}} \times 100 \end{eqnarray*}

### Statistical analysis

Data was analyzed using analysis of variance while mean values were compared using Fisher’s least significant difference (LSD) tests at 5% probability level (Steel, Torrie & Deekey, 1997).

## Results

### Germination rate

**Figure 1:**
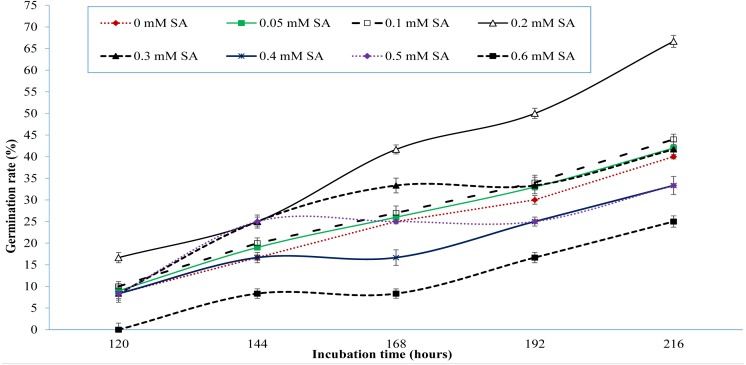
Germination response of sweet pepper cv. Yolo wonder to salicylic acid under salt-stress of 60 mM NaCl (Lab study).

Monitoring of seed germination at interval of every 24 h disclosed that signs of germination appeared at 120 h of incubation. Figure 1 shows variations in seed germination from 120 to 216 h of incubation. It was observed that salinity of 60 mM NaCl severely affected germination of seed in control treatment. The reported 85% germination of sweet pepper cv. Yolo wonder by seed supplier was reduced to 75% due to salinity of 60 mM NaCl (Table 1, Fig. 1). However, treatments of SA showed an increase in germination rate even under salinity of 60 mM NaCl (Fig. 1). Figure 1 also depicts that the seed germination improved gradually with increasing units of SA till 0.3 mM SA but onward application of SA i.e., 0.4, 0.5 and 0.6 mM SA inhibited the seed germination (Fig. 1). Treatment of 0.2 and 0.3 mM SA appeared as optimal dose as all seeds of these treatments germinated up to 312 h of incubation, compared to control treatment (Table 1).

### Morph physiological characters

Salinity significantly reduced the morphological characteristics of seedlings such as length, biomass, leaf area, chlorophyll contents etc. in control treatment (Table 1). Contrary to control, the seedlings in SA treatments were more vigorous, longer in root and shoot length showing no symptoms of salinity stress (Table 1). However, changes in growth parameters of seedling varied with variations in exogenous SA rates. Application of 0.05 to 0.3 mM SA ameliorated 60 mM NaCl injury on seedling growth and vigor among SA treatments. These rates of SA increased biomass, root and shoot length of seedling by 15–20% in each, respectively compared to control treatment under salinity of 60 mM NaCl (Table 1). However, SA >0.3 mM suppressed the growth and gradually reduced biomass, root and shoot length with gradual increase in SA concentration (Table 1). It was concluded that application of SA upto 0.3 mM might be significantly reduced the detrimental effect of salinity induced by 60 mM NaCl.

A significant reduction in plant growth and yield occurred in control treatment due to salinity of 60 mM NaCl. The plants of control treatment were stunted with less number of branches, leaves, flowers, and fruits per plant, etc (Table 2). Salinity also severely affected leaf development of these plants and thus very low values of leaf area were observed in these plants. Contrary to it, the plants of SA treatments showed significant improvement in the aforementioned growth variables due to the physiological role of SA under salinity stress. Application of 0.3 mM SA appeared to be an optimal concentration of SA for sweet pepper in order to ameliorate salinity stress on its growth and yield (Table 2). Exogenous SA effectively combated salinity stress in sweet pepper and thus SA treated plants showed an increase in branches per plant by 5–15%, leaves per plant by 10–30%, leaf area by 9–25%, number of flowers per plant by 8–24% and finally fruits per plant by 3–18% (Table 2). But, reductions in these parameters were observed in plants treated by SA > 0.3 mM (Table 2).

**Table 1:** Comparative effects of different concentrations of salicylic acid on seedling growth at 312 h after incubation under salinity stress (*n* = 4). Values in column sharing same superscript letter(s) do not differ at *p* = 0.05 according to LSD test.

**SA rate(mM)**	**Seed germination(%)**	**Length (cm)**		**Biomass** (**mg plate**^−1^)
		**Root**	**Shoot**	
0	75.0 ± 2.1^d^	1.53 ± 0.12^b^	1.26 ± 0.11^ab^	44.7 ± 1.9^c^
0.05	83.3 ± 1.5^c^	1.54 ± 0.04^b^	1.28 ± 0.11^ab^	47.0 ± 1.81^bc^
0.1	91.7 ± 1.1^b^	1.62 ± 0.09^ab^	1.32 ± 0.11^ab^	52.6 ± 1.2^b^
0.2	100 ± 0^a^	1.70 ± 0.06^ab^	1.38 ± 0.13^ab^	53.7 ± 1.5^b^
0.3	100 ± 0^a^	1.74 ± 0.08^ab^	1.5 ± 0.12^a^	51.9 ± 1.1^bc^
0.4	91.7 ± 1.1^b^	1.83 ± 0.09^a^	1.55 ± 0.12^a^	69.6 ± 1.0^a^
0.5	91.7 ± 1.1^b^	1.25 ± 0.12^c^	1.24 ± 0.13^ab^	51.5 ± 0.52^bc^
0.6	61.0 ± 2.1^e^	1.10 ± 0.05^c^	1.14 ± 0.03^b^	48.3 ± 2.5^bc^

**Figure 2:**
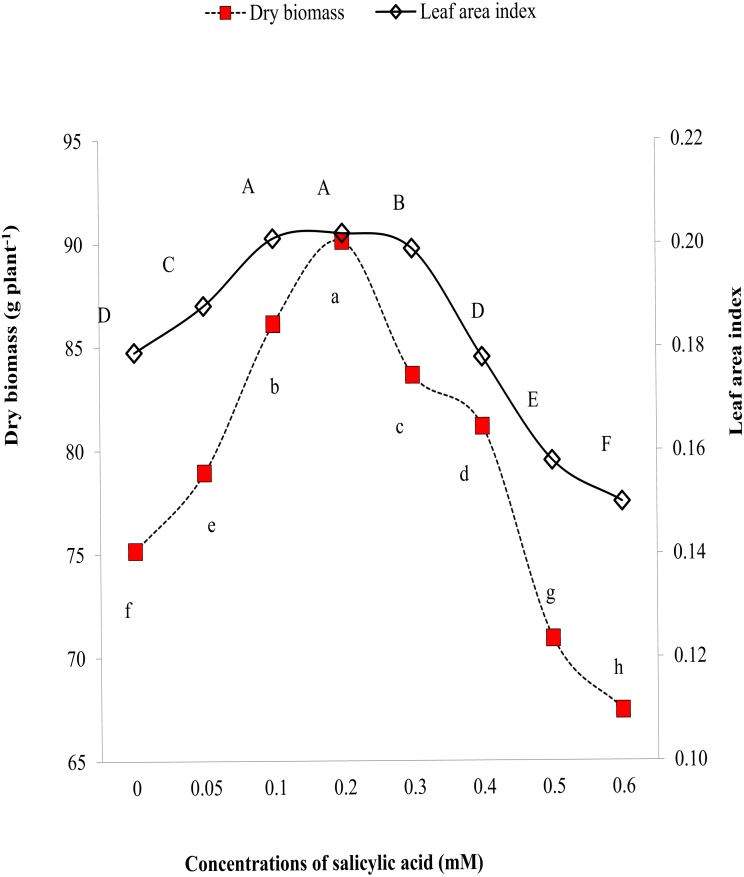
Comparative effects of different concentrations of salicylic acid on biomass and leaf area index of sweet pepper under salinity stress. Markers with same letter(s) do not differ at *p* = 0.05 according to LSD test. SA, Salicylic acid LSD for shoot dry weight = 0.4797, LSD for LAI = 0.00115.

Leaf area index and dry biomass are effective indicators of morphological characteristics. Higher values of LAI and biomass reflect improvements in morphological characteristics of plants. Results explored that salinity significantly reduced LAI and biomass but exogenous SA reversed these changes (Fig. 2). The highest LAI and biomass per plant were found in plants treated by 0.3 mM SA (Fig. 2). Figure 2 also revealed gradual increase in LAI and biomass with an increment of SA till 0.3 mM and suppression in LAI and biomass on application of SA > 0.3 mM doses (Fig. 2).

**Table 2:** Comparative effects of different concentrations of salicylic acid on vegetative growth of sweet pepper under salinity stress (*n* = 3). Values in column sharing same letter(s) do not differ at *p* = 0.05 according to LSD test.

**SA rate(mM)**	**Branches plant**^−1^	**Leaves plant**^−1^	**Leaf area** (**cm**^**2**^**leaf**^−1^)	**Flowersplant**^−1^	**Fruitsplant**^−1^
0	7.5 ± 0.15^d^	100 ± 2.0^d^	24.0 ± 0.48^d^	31.3 ± 0.62^d^	10.4 ± 0.21^d^
0.05	7.8 ± 0.15^cd^	104 ± 2.0^cd^	25.1 ± 0.50^cd^	32.7 ± 0.65^cd^	10.8 ± 0.22^cd^
0.1	8.2 ± 0.16^bc^	110 ± 2.2^bc^	26.4 ± 0.52^bc^	34.3 ± 0.68^bc^	11.4 ± 0.23^bc^
0.2	8.4 ± 0.17^ab^	112 ± 2.2^ab^	26.9 ± 0.54^ab^	35.2 ± 0.70^ab^	11.7 ± 0.23^ab^
0.3	8.9 ± 0.18^a^	119 ± 2.4^a^	28.7 ± 0.57^a^	37.4 ± 0.74^a^	12.4 ± 0.25^a^
0.4	8.1 ± 0.16^bc^	108 ± 2.4^bc^	25.9 ± 0.52^bc^	33.7 ± 0.67^bc^	11.2 ± 0.22^bc^
0.5	6.2 ± 0.12^e^	84 ± 1.7^e^	20.1 ± 0.40^e^	26.1 ± 0.52^e^	8.6 ± 0.17^e^
0.6	5.7 ± 0.12^e^	76 ± 1.5^e^	18.4 ± 0.37^e^	24.0 ± 0.48^e^	7.9 ± 0.16^e^

### SA based ethylene regulation and electrolyte leakage

Different researchers reported dramatic increases in ethylene biosynthesis in plants under salinity stress (Morgan & Drew, 1997). However, ethylene can be regulated through endogenous SA. Salicylic acid regulates ethylene in plants by two ways; (i) Firstly by suppressing ethylene forming enzyme (EFE) and/or ACC synthase (Mattoo & Suttle, 1991; Yang & Hoffman, 1984) and (ii) Secondly by triggering biosynthesis of spermine, spermidine and putrescine and/or polyamines from SAM (Mittler et al., 2011; Freschi, 2013).

The data related to ethylene evolution from seedlings and endogenous SA is presented in Table 3. Results reveal that maximum ethylene evolution (3.19 nmole plate^−1^) was found in control treatment, showing comparatively more endogenous ethylene biosynthesis. But contrary to control treatment, ethylene evolution from SA treated treatments ranged from 2.23 to 3.12 nmole plate^−1^ (Table 3). Less ethylene evolution in SA treatments reveal the suppression of endogenous ethylene biosynthesis due to physiological role of SA reported under abiotic stresses (Table 3).

Electrolyte leakage is considered as one of the most brutal effects of abiotic stresses particularly of salinity stress due to physiological drought and specific ions toxicity. Significant variations in EL were observed due to varying SA treatments compared to control treatment under salinity stress. The least EL was found in treatment of 0.2 mM SA, followed by 0.3 mM SA (Table 3).

**Figure 3:**
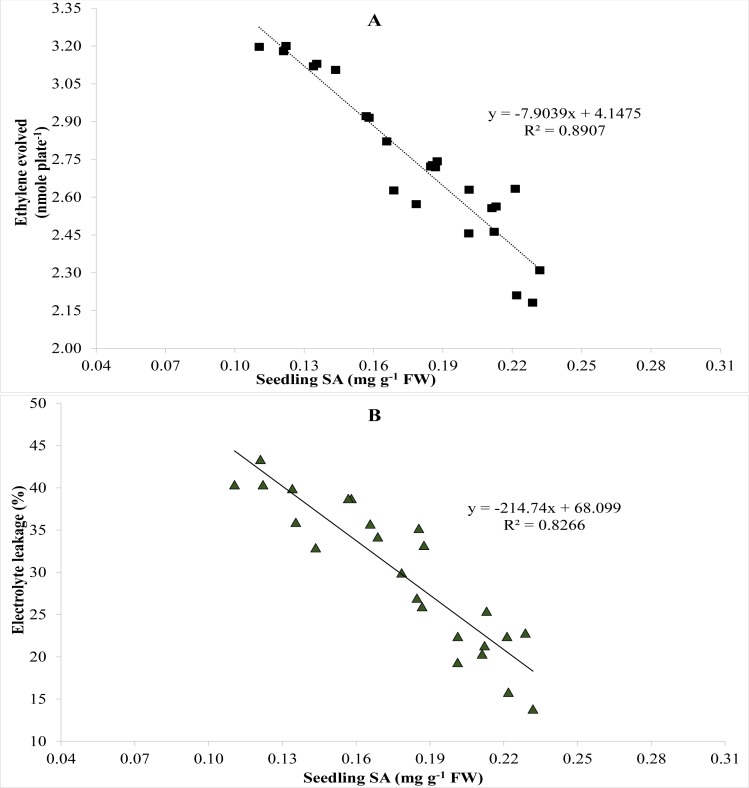
Pearson correlation between (A) salicylic acid and ethylene evolved and (B) salicylic acid and electrolyte leakage (*n* = 4).

**Figure 4:**
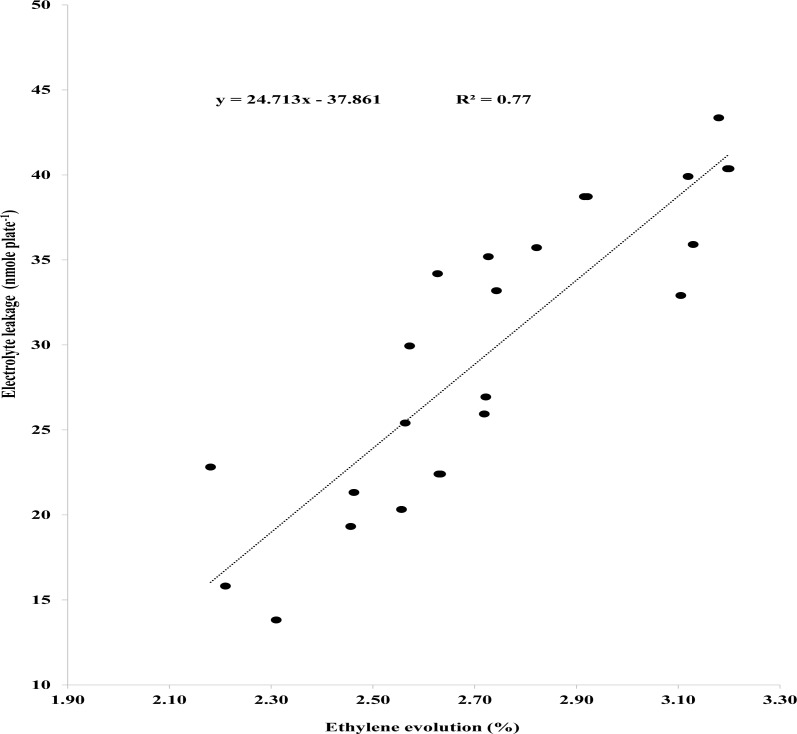
Pearson correlation between ethylene evolution and electrolyte leakage. Bars show standard errors (*n* = 4).

A relationship among EL, endogenous SA in seedlings and ethylene was established using Pearson’s correlation. Pearson’s correlation showed negative correlation between ethylene evolved and endogenous SA in seedlings (Fig. 3A). Ethylene biosynthesis dropped steadily as endogenous SA escalated (Fig. 3A). Likewise, Person’s correlation also indicates a negative relationship between endogenous SA in seedlings and EL (Fig. 3B), while a positive correlation between ethylene evolution and EL (Fig. 4).

**Table 3:** Impacts of salicylic acid on ethylene evolution, leaf SA contents and electrolyte leakage in sweet pepper at 312 h after incubation under salinity stress (*n* = 3). Values in column sharing same superscript letter(s) do not differ at *p* = 0.05 according to LSD test.

**SA rate(mM)**	**Ethylene evolution** (**mg plate**^−1^)	**Leaf SA contents** (**mg g**^−1^**FW**)	**Electrolyte leakage(El)(%)**
0	3.19 ± 0.01^a^	0.12 ± 0.003^f^	39.9 ± 0.82^a^
0.05	3.12 ± 0.01^a^	0.14 ± 0.003^e^	35.2 ± 1.66^b^
0.1	2.89 ± 0.03^b^	0.16 ± 0.003^d^	25.9 ± 0.82^c^
0.2	2.70 ± 0.03^c^	0.18 ± 0.006^c^	20.8 ± 0.47^d^
0.3	2.67 ± 0.04^c^	0.18 ± 0.002^c^	21.3 ± 0.98^d^
0.4	2.61 ± 0.02^c^	0.21 ± 0.006^b^	22.4 ± 0.82^d^
0.5	2.49 ± 0.03^d^	0.20 ± 0.004^b^	38.7 ± 0.47^a^
0.6	2.23 ± 0.03^e^	0.23 ± 0.003^a^	40.4 ± 2.23^a^

### Photosynthetic activity and water use efficiency

Salinity directly affected photosynthetic machinery in sweet pepper by inducing physiological drought stress. Soil salinity of 60 mM NaCl brutally affected net assimilation rate, stomatal conductance, and transpiration rate. The photosynthetic rate was observed 11.84 µmol m^−2^ s^−1^ in control which increased with application of 0.05 to 0.3 mM SA to 15.09 µmol m^−2^ s^−1^ (Table 4). It was also observed that the application of SA ≥ 0.4 mM reduced photosynthetic rate. The similar effect of SA application was observed on Stomatal conductance and transpiration rate in sweet pepper. Maximum transpiration (3.98 nmole m^−1^ s^−1^) was recorded in plants without SA application which gradually decreased to 2.91 nmole m^−1^ s^−1^ with gradual increase in SA concentration (Table 4). Likewise, salicylic acid also positively regulated the stomatal conductance, indicating physiological improvement of photosynthetic machinery under salinity stress.

Salinity reduced instantaneous WUE due to the osmotic effect of 60 mM NaCl in control treatment (Table 4). Data related to instantaneous WUE discloses that plants treated SA had more instantaneous WUE than untreated plants (Table 4). The treatment of 0.05–0.3 mM SA was found more effective than others on the basis of instantaneous WUE in sweet pepper under salinity stress.

**Figure 5:**
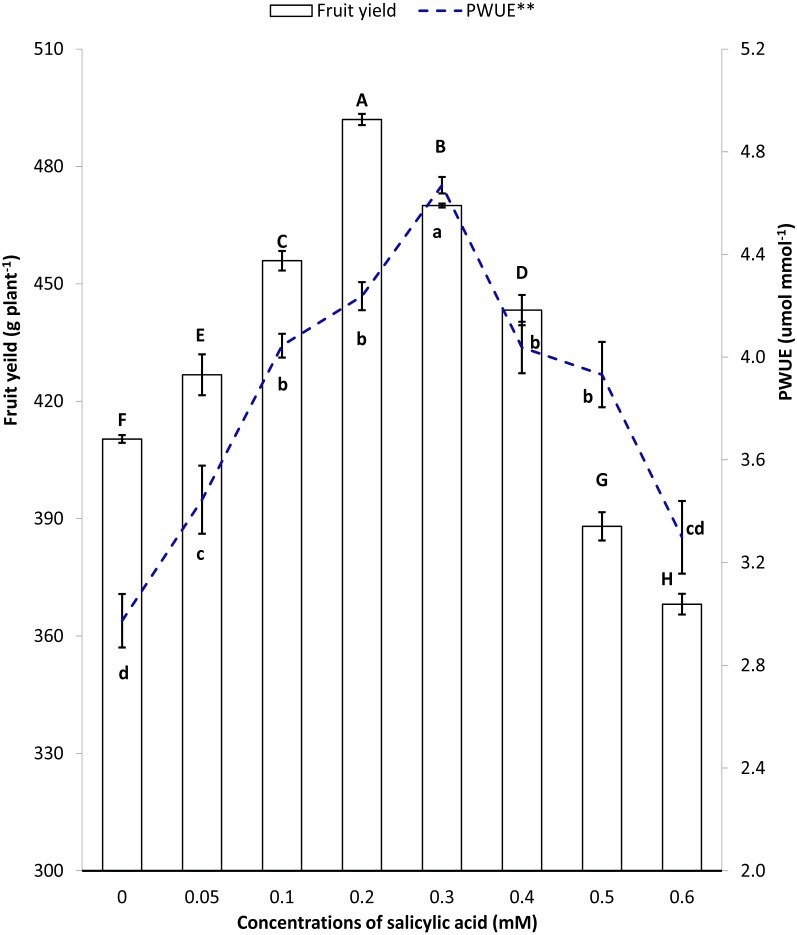
Pearson’ correlation between fruit yield and instantaneous water use efficiency under salinity stress. **Instantaneous water use efficiency, SA, Salicylic acid. Markers with same letter(s) do not differ at *p* = 0.05 according to LSD test (LSD value = 0.0679). Bars with same letter(s) do not differ at *p* = 0.05 according to LSD test (LSD value = 2.6179).

Figure 5 collectively presents the trend of improvements in fruit yield and instantaneous WUE under salinity stress. Compared to control, plants producing higher fruit yield per plant had corresponding higher instantaneous WUE in SA treatments (Fig. 5).

### Biochemical attributes regulation by SA

Salinity generally cracks down defensive system of plants either by hormonal imbalance or by inducing oxidative stress. Salicylic acid is one of those plant growth regulators that regulate antioxidant enzymes under salinity stress. It was observed that SA alleviated salt injurious effects on germination, morphological and physiological characters of sweet pepper by regulating SOD, POD, and TSS. Results of lab study revealed that salicylic acid was also protected chlorophyll a + b from injurious effects of salinity and thus led toward more accumulation of chlorophyll a + b in leaves of treated seedlings compared to control (Table 5). Among different tested rates, 0.2–0.3 mM SA were found more effective for improving chlorophyll contents (Table 5). Table 5 also depicts that SA treated seedlings showed higher SOD activity with high TSS values compared to untreated seedlings.

**Figure 6:**
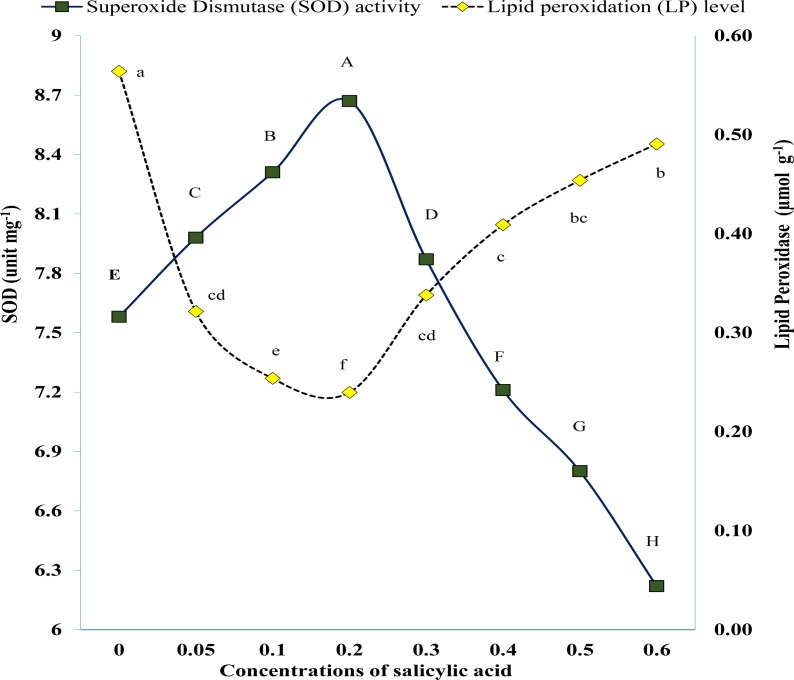
Variations in antioxidant enzymes as result of foliar application of salicylic acid under salinity stress. Markers with same letter(s) do not differ at *p* = 0.05 according to LSD test. SA, Salicylic acid LSD for SOD = 0.0404, LSD for LP = 0.000423.

Results of pot study revealed that exogenous SA significantly affected the activity of SOD and POD under salinity of 60 mM NaCl (Fig. 6). The POD activity was significantly higher in untreated plants (control) while lower in SA treated plants (Fig. 6). The POD activity first gradually reduced with increment of SA concentration till 0.3 mM. Afterwards it was started to rise (Fig. 6). Similarly, SOD activity was low in plants of control treatment which was improved significantly in plants where salicylic acid was applied. The SOD activity gradually improved with the increasing rate of SA but up to 0.3 mM (Fig. 6).

### Fertilizer use efficiency

Soil salinity had strong effect on K^+^ use efficiency as Na^+^ ions competes K^+^ ions for major binding sites in key metabolic processes in the cytoplasm and inhibits >50 enzymes activity, being activated by K^+^. In nutshell, K deficiency results in decrease in photosynthetic CO_2_ fixation and assimilates transport and utilization. Resultantly, lower K use efficiency (agronomic and recovery) was found in plants of control treatments (Table 6). SA treatments significantly improved K use efficiency by ameliorating salinity stress. However, the decrease in K content is due to K-efflux induced by Na^+^ (Bojórquez-Quintal et al., 2014).

**Table 4:** Variations in photosynthetic activities and water use efficiency in sweet pepper as influenced by foliar application of salicylic acid under salinity stress (*n* = 3). Values in column sharing same superscript letter(s) do not differ at *p* = 0.05 according to LSD test.

**SA rate(mM)**	**A**_N_ (**µmol m**^−2^**s**^−1^)	**Gs** (**mol m**^−2^**s**^−1^)	**E** (**mmol m**^−1^**s**^−1^)	**PWUE** (**µmol mmol**^−1^)
0	11.84 ± 0.44^d^	0.377 ± 0.05^a^	3.98 ± 0.02^a^	2.97 ± 0.10^d^
0.05	12.30 ± 052^d^	0.323 ± 0.04^ab^	3.57 ± 0.02^b^	3.44 ± 0.13^c^
0.1	13.59 ± 0.22^bc^	0.290 ± 0.04^ac^	3.36 ± 0.02^c^	4.04 ± 0.05^b^
0.2	13.91 ± 0.24^ab^	0.283 ± 0.01^ac^	3.28 ± 0.02^cd^	4.24 ± 0.05^b^
0.3	15.09 ± 0.17^a^	0.250 ± 0.04^bc^	3.23 ± 0.02^d^	4.67 ± 0.03^a^
0.4	12.59 ± 0.25^cd^	0.233 ± 0.02^bc^	3.12 ± 0.01^e^	4.04 ± 0.10^b^
0.5	11.99 ± 0.40^d^	0.217 ± 0.01^bc^	3.05 ± 0.01^e^	3.93 ± 0.13^b^
0.6	9.58 ± 0.19^e^	0.187 ± 0.01^c^	2.91 ± 0.07^f^	3.30 ± 0.14^cd^

**Notes.**

A_N_ Net photosynthetic rate Gs Stomatal conductance E transpiration PWUE Photosynthetic water use efficiency

**Table 5:** Impacts of salicylic acid on total soluble sugars, SOD activity and chlorophyll (a & b) contents in sweet pepper seedlings at 312 h after incubation under salinity stress (*n* = 4). Values in column sharing same superscript letter(s) do not differ at *p* = 0.05 according to LSD test.

**SA rate(mM)**	**Total soluble sugars** (**mg g**^−1^**FW**)	**SOD** (**activity h**^−1^**100 mg**^−1^)	**Chlorophylla & b** (**µg g**^−1^)
0	13.7 ± 0.56^bc^	4.64 ± 0.19^cd^	89 ± 2.1^ab^
0.05	14.3 ± 0.58^ab^	4.82 ± 0.20^b−d^	93 ± 2.2^ab^
0.1	15.1 ± 0.62^a^	5.01 ± 0.21^a−c^	95 ± 2.3^ab^
0.2	14.3 ± 0.58^ab^	5.19 ± 0.21^ab^	99 ± 2.2^ab^
0.3	15.0 ± 0.61^ab^	5.38 ± 0.22^a^	102 ± 2.3^a^
0.4	12.9 ± 0.53^cd^	4.87 ± 0.20^b−d^	110 ± 2.0^a^
0.5	12.1 ± 0.49^de^	4.45 ± 0.18^de^	58 ± 1.8^bc^
0.6	11.5 ± 0.47^e^	4.13 ± 0.17^e^	35 ± 1.7^c^

Salinity also had a strong effect on N and P use efficiency. But SA improved N and P use efficiency by ameliorating salinity stress (Table 6). Table 6 clearly depicts that agronomic use efficiency of N, P and K was 54, 99 and 89 g g^−1^, respectively in control due to strong effect of salinity which increased up to 117, 212 and 190 g g^−1^ due to SA application @ 0.3 mM, respectively.

## Discussion

Salicylic acid potentially regulated physiology and morphology of germinated seeds and plants to alleviate effects of salinity stress. Greenberg, Silverman & Liang (2000) and Nemeth et al. (2002) also reported similar findings. It has been reported that salinity stress dramatically increased ethylene biosynthesis in plants (Morgan & Drew, 1997; Tao et al., 2015) which was reduced due to salicylic acid even in the atmosphere of salinity stress. Similar findings were also reported by Shakirova et al. (2003), Patil & Gaikwad (2011) and Anwar et al. (2013) under salts stressed conditions. Salinity reduced seed germination by triggering ethylene synthesis at alarming rate. Higher ethylene evolution was observed in control treatment compared with SA treatments (Table 3). In our study, ethylene was found higher in control which reduced due to the gradual increase in leaf salicylic acid contents (Fig. 3A) and thus led to elevated germination percentage and better seedling growth due to improvement in physiological and morphological characteristics. Reduction in ethylene biosynthesis has also been reported by Leslie & Romani (1988) and Quiroz-Figueroa et al. (2001) after the addition of SA. Our results shown in Fig. 3 in terms of correlation between ethylene evolution and leaf SA contents are also in accordance with findings of Leslie & Romani (1988) and Quiroz-Figueroa et al. (2001). Seed priming with SA improved seed germination percentage and seedling growth under saline environment (Fig. 1; Table 1).

**Table 6:** Variations in fertilizer use and recovery efficiency in sweet pepper as influenced by foliar application of salicylic acid under salinity stress (*n* = 3). Values in column sharing same superscript letter(s) do not differ at *p* = 0.05 according to LSD test.

**Salicylic acid(mM)**	**Agronomic nutrient use efficiency** (**g g**^−1^**FW** )			**Recovery efficiency(%)**		
	**Nitrogen**	**Phosphorus**	**Potassium**	**Nitrogen**	**Phosphorus**	**Potassium**
0	54 ± 0.38^f^	99 ± 0.69^f^	89 ± 0.62^f^	33.6 ± 1.94^c^	21.9 ± 0.37^d^	42 ± 3.05^c^
0.05	67 ± 3.63^e^	121 ± 6.65^e^	109 ± 5.90^e^	34.5 ± 0.55^c^	22.5 ± 0.15^cd^	44 ± 2.14^c^
0.1	89 ± 1.15^c^	162 ± 2.07^c^	145 ± 1.86^c^	40.2 ± 0.49^bc^	24.3 ± 0.22^bc^	50 ± 1.93^bc^
0.2	117 ± 2.13^a^	212 ± 3.84^a^	190 ± 3.46^a^	44.8 ± 1.20^ab^	26.1 ± 0.25^b^	57 ± 2.06^b^
0.3	100 ± 0.92^b^	181 ± 1.66^b^	163 ± 1.49^b^	50.8 ± 0.267^a^	29.7 ± 0.48^a^	69 ± 3.35^a^
0.4	79 ± 2.26^d^	144 ± 4.73^d^	129 ± 4.25^d^	36.8 ± 4.19^c^	24.3 ± 0.156^bc^	50 ± 6.93^bc^
0.5	37 ± 3.42^g^	68 ± 6.18^g^	61 ± 5.56^g^	10.3 ± 1.02^d^	12.9 ± 0.32^e^	10 ± 1.08^d^
0.6	22 ± 1.51^h^	40 ± 2.73^h^	36 ± 2.46^h^	7.3 ± 0.35^d^	9.6 ± 0.30^f^	−2 ± 1.17^d^

Improvement in morphological and physiological characteristics particularly leaf area, LAI, flowering, fruiting (Table 2), photosynthesis, stomatal conduction, transpiration or water use efficiency (Table 4) might be due to the physiological role of salicylic acid as discussed earlier. These improvements occurred due to less ethylene biosynthesis under controlled conditions as similar results have also been reported earlier by Metwally et al. (2003), Cevahir et al. (2006) and Kazemi & Shokri (2011) who found in delay in senescence -a critical feature of ethylene upon application of salicylic acid. As the application of salicylic acid suppressed the ethylene evolution from the imbibed seed under salt stress and a positive correlation exists among ethylene concentration in plate-air and leaf salicylic acid, it can be concluded that salicylic acid improved plant growth and yield even under stressed conditions just by working via reduction on ethylene synthesis. Keeping in view the biochemical characteristics, the possible explanation for these changes might be increased K use efficiency (Table 6) due to SA based K-efflux and membrane depolarization (Jayakannan et al., 2013). This phenomenon gained even more importance when salinity itself caused a decrease in chlorophyll pigments, SOD activity and total soluble sugars in seedlings while net photosynthetic rate, WUE, SOD activity and low N, P and K use efficiency in plants of sweet pepper. Owing to salicylic acid, increase in soluble proteins, total soluble sugars, total phenolic and chlorophyll pigments have been also reported in chickpea seeds under normal and stressed conditions by Patel & Krishnamurthy (2013) and Kabiri, Nasibi & Farahbakhsh (2014). Recently reported work of Khan et al. (2019) also supports our findings. Khan et al. (2019) also reported that exogenous SA ameliorated salinity stress in rice (*Oryza sativa* L.). The amelioration of salinity effects on plants might be due to activation of antioxidant mechanisms and gene expression as reported by El-Esawi et al. (2017). However, plants can escape from such kinds of stresses by developing a defense system comprising antioxidant enzymes like SOD, CAT, and POD (Borsani, Valpuesta & Botella, 2001). A number of factors mediate the production of these antioxidants and exogenous SA is one of them. In this study, besides osmotic adjustments, salicylic acid significantly increased SOD activity in seedlings (Table 5) and plant tissues with a decrease in lipid peroxidase which is the second probability of improved physio-morphological characteristics of seed germinated and plants of sweet pepper under salt-stressed conditions. Patel & Krishnamurthy (2013) summed up both positive and negative effects of salicylic acid on enzyme activities in plant tissues. In accordance to our results, Kabiri, Nasibi & Farahbakhsh (2014) reported SA induced increase in activities of CAT, APX, and GPX in plants of *Nigella sativa*, which led to reduction in H_2_O_2_ content, lipid peroxidation (MDA) and LOX activity. Exogenous SA significantly ameliorated salinity stress by elevating antioxidant activities, proteins contents and MDA (Cag et al., 2009; El-Esawi et al., 2017). Similar to our results, Kabiri, Nasibi & Farahbakhsh (2014) reported that different concentrations of salicylic acid had a significant correlation with SA induced EL and enzymatic activities.

Salicylic acid-mediated production of antioxidant enzymes might be due to its singling phenomenon or triggering stress proteins and explained by Pandey et al. (2000), Borsani, Valpuesta & Botella (2001), Mittler et al. (2011) and Freschi (2013). Kabiri, Nasibi & Farahbakhsh (2014) showed a very close correlation between enzymatic activities and salicylic acid in improving seed germination, growth, and fruiting under salt stress by reducing ROS. Interestingly, the application of salicylic acid significantly reduced lipid peroxidation (ROS representative) in this study (Fig. 6) and increased SOD (Fig. 6) contents in salt-stressed plant tissues. Moreover, our results are in agreement with findings of Cag et al. (2009) and Joseph, Jini & Sujatha (2010).

The increased K use efficiency (Table 6) might be due to signaling of SA. SA is reported to ameliorate salinity stress by restoring membrane potential and decreasing K^+^ efflux via GORK channels (Jayakannan et al., 2013). Increased N use efficiency might be due to SA as it is reported to increase N fixation and N assimilation by stabilizing membrane structure and fluidity (Hayat et al., 2014). Synergetic effects of K with N and P might be reason for improved N, and P use efficiency under salinity stress.

## Conclusion

Salinity induced by 60 mM NaCl strongly triggered ethylene evolution in bell pepper seedlings with significant electrolyte leakage. Seedlings treated with SA elucidated that ethylene evolution from seedlings was reduced where leaf SA contents were higher compared to control treatment. Moreover, pre-treatment with 0.3 mM SA ameliorated salinity effect of 60 mM NaCl by optimizing ethylene level, up-lifting TSS, chlorophyll contents with higher SOD activity. Our results suggest that SA pretreatment can be a promising tool for ethylene homeostasis to salinity tolerance in bell pepper. In future, the research on interactions of SA with other growth regulators will give more insights for ameliorating effects of salinity on bell pepper.

##  Supplemental Information

10.7717/peerj.8475/supp-1 Data S1 Experimental raw data

## Additional Information and Declarations

### Data Availability

The following information was supplied regarding data availability: The raw data is available as a Supplemental File.
